# Prognostic value of Diabetes in Patients with Nasopharyngeal Carcinoma Treated with Intensity-Modulated Radiation Therapy

**DOI:** 10.1038/srep22200

**Published:** 2016-03-01

**Authors:** Hao Peng, Lei Chen, Yuan Zhang, Wen-Fei Li, Yan-Ping Mao, Fan Zhang, Rui Guo, Li-Zhi Liu, Ai-Hua Lin, Ying Sun, Jun Ma

**Affiliations:** 1 Department of Radiation Oncology, Sun Yat-sen University Cancer Center, State Key Laboratory of Oncology in Southern China, Collaborative Innovation Center for Cancer Medicine, People’s Republic of China; 2 Imaging Diagnosis and Interventional Center, Sun Yat-sen University Cancer Center, State Key Laboratory of Oncology in Southern China, Collaborative Innovation Center for Cancer Medicine, People’s Republic of China; 3 Department of Medical Statistics and Epidemiology, School of Public Health, Sun Yat-sen University, People’s Republic of China

## Abstract

The prognostic value of diabetes remains unknown in nasopharyngeal carcinoma (NPC) treated with intensity-modulated radiation therapy (IMRT). We retrospectively reviewed medical records of 1489 patients with non-metastatic, histologically-proven NPC treated using IMRT. 81/1489 (5.4%) patients were diabetic, 168/1489 (11.3%) were prediabetic, and 1240/1489 (83.3%) were normoglycemic. The 4-year disease-free survival (DFS), overall survival (OS), loco-regional relapse-free survival (LRRFS) and distant metastasis-free survival (DMFS) rates were 77.1% vs. 82.4% (*P* = *0.358*), 85.8% vs. 91.0% (*P* = 0.123), 90.9% vs. 91.7% (*P* = 0.884), and 85.5% vs. 89.2% (*P* = 0.445) for diabetic vs. normoglycemic patients, and 82.4% vs. 82.4% (*P* = 0.993), 88.7% vs. 91.0% (*P* = 0.285), 90.6% vs. 91.7% (*P* = 0.832) and 91.5% vs. 89.2% (*P* = 0.594) for preidabetic vs. normoglycemic patients. Multivariate analysis did not established diabetes as poor prognostic factors in NPC patients treated with IMRT (*P* = 0.332 for DFS, *P* = 0.944 for OS, *P* = 0.977 for LRRFS, *P* = 0.157 for DMFS), however, triglycerides and low density lipoprotein cholesterol were independent prognostic factors. In conclusion, diabetes does not appear to be a prognostic factor in NPC patients treated with IMRT, and attention should be paid to hyperglycemia-associated hyperlipaemia.

Data on global cancer revealed an estimated 84,400 new cases of nasopharyngeal carcinoma (NPC) and 51,600 deaths in 2011^1^. Nasopharyngeal carcinoma (NPC) is a cancer with an unbalanced geographical distribution, with an age-standardised incidence rate of 20–50 per 100,000 males in south China but only 0·5 per 100,000 in predominantly white populations of European origin^1^. Due to anatomic constraints and a high degree of radiosensitivity, radiotherapy is the main treatment for NPC, and the TNM staging system is the most reliable method for devising clinical treatment strategies and predicting prognosis^2^. However, the TNM staging system is based solely on anatomy and is therefore not particularly accurate for risk segregation and survival prediction^3^, and other prognostic factors such as Epstein-Burr virus (EBV) DNA^4^,^5^, primary tumour volume^6^,^7^ and pretreatment serum lactate dehydrogenase (LDH) levels are used^8^.

Comorbidity in patients with diabetes and certain types of cancer is not uncommon^9^,^10^, and people with diabetes have an elevated risk of cancers of the liver, biliary tract, pancreas, stomach, colorectum, kidney, bladder, breast and endometrium^11^,^12^,^13^,^14^. Additionally, lung cancer^15^, pancreatic cancer^16^, breast cancer^17^,^18^ and head and neck cancer^19^ patients with elevated blood glucose have poorer prognosis than normoglycemic patients.

The prognostic value of diabetes in NPC patients remains controversial. A positive outcome was reported for NPC patients treated with 2-dimensional (2D) radiotherapy in one study^20^, but others found that diabetes has no prognostic value in patients treated with 2D radiotherapy and intensity-modulated radiation therapy (IMRT)^21^. Importantly, neither study investigated the relationship between diabetes and plasma Esptein-Barr virus (EBV) DNA, which has been proven to be a reliable molecular marker for guiding treatment and predicting prognosis^4^,^5^,^22^. The prognostic value of diabetes in NPC patients treated with IMRT should therefore be re-evaluated to identify other more useful prognosis-related factors. In the present study, we conducted a retrospective analysis of existing patient data to explore the long-term prognostic impact of diabetes on the outcome of improved radiotherapy treatment in NPC patients.

## Methods and Materials

### Patient selection

We retrospectively analysed data from 1811 patients with previously untreated, biopsy-confirmed NPC that showed no evidence of distant metastasis between November 2009 and February 2012 at Sun Yat-sen University Cancer Center. 322 patients were excluded due to a lack of blood glucose and pre-DNA data, which left 1489 patients for further study. All experimental protocols in this retrospective study were approved by the Research Ethic Committee of Sun Yat-sen University Cancer Center, confidential patient information was protected at all times, and informed consent was obtained from all patients.

### Clinical staging

All the methods in this current study were carried out in accordance with the approved guidelines. The routine staging process included a complete medical history and clinical examination of the head and neck region, direct fiber-optic nasopharyngoscopy, magnetic resonance imaging (MRI) of the skull base and the entire neck, chest radiography, a whole-body bone scan, abdominal sonography and positron emission tomography (PET)-CT. Tumour-associated markers immunoglobulin A (IgA) antibodies to EBV viral capsid antigen (VCA) and to EBV early antigen (EA) were tested, along with plasma EBV DNA. All patients had a dental evaluation before radiotherapy and were restaged according to the 7^th^ edition of the International Union against Cancer/American Joint Committee on Cancer (UICC/AJCC) system^23^. All MRI materials and clinical records were reviewed to minimize heterogeneity in restaging. Two radiologists employed at our hospital separately evaluated all of the scans and disagreements were resolved by consensus.

### Real-time quantitative EBV DNA PCR

Measurement of the plasma EBV DNA level was made before treatment, and plasma DNA was extracted and assayed using real-time quantitative PCR as described previously^24^. The real-time quantitative PCR system was developed for plasma EBV DNA detection, and targeted the *BamH*I-W region of the EBV genome using primers 5′-GCCAGAGGTAAGTGGACTTT-3′ and 5′-TACCACCTCCTCTTCTTGCT-3′. The dual fluorescence-labelled oligomer 5′-(FAM)CACACCCAGGCACACACTACACAT(TAMRA)-3′ served as a probe. Sequence data for the EBV genome were obtained from the GeneBank sequence database.

### Diagnosis of diabetes and prediabetes

All patient medical records were thoroughly reviewed, including pre-treatment biochemical profile, medical history, and daily fasting plasma glucose (FPG) levels before and after meals during treatment if the patients had these data. According to the 2015 diagnosis and classification of diabetes mellitus by American Diabetes Association (ADA)^25^, FPG and 2-hour plasma glucose after a 75 g oral glucose load (2hPG) at baseline were measured. Patients were classified into diabetic (FPG ≥7.0 mmol/L and 2hPG ≥11.1 mmol/L), prediabetic (FPG between 6.0 and 7.0 mmol/L and 2hPG between 7.8 and 11.1 mmol/L) and normoglycemic (FPG ≤6.0 mmol/L) groups.

### Treatment

#### Radiotherapy

All patients were treated with definitive IMRT at Sun Yat-sen University Cancer Center. Immobilization was carried out with a custom-made thermoplastic cast from head-to-neck with the patient resting on a neck support. A high-resolution planning computed tomography scan (Siemens, Plus 4) with contrast was taken from the vertex down to 2 cm below the sternoclavicular joint, and each slice was 3 mm. Target volumes were delineated slice-by-slice on treatment planning CT scans using an individualized delineation protocol that complies with International Commission on Radiation Units and Measurements reports 50 and 62. The prescribed doses were 66–72 Gy at 2.12–2.43 Gy/fraction to the planning target volume (PTV) of the primary gross tumour volume (GTVnx), 64–70 Gy to the PTV of the GTV of the involved lymph nodes (GTVnd), 60–63 Gy to the PTV of the high-risk clinical target volume (CTV1), and 54–56 Gy to the PTV of the low-risk clinical target volume (CTV2). All targets were treated simultaneously using the simultaneous integrated boost technique.

#### Chemotherapy

Before treatment, we recommended radiotherapy alone for stage I patients, concurrent chemoradiotherapy for stage II patients, and concurrent chemoradiotherapy (CCRT) +/− neoadjuvant/adjuvant chemotherapy for stage III to IVA-B patients, according to our institutional guidelines. Neoadjuvant chemotherapy was given mainly when the waiting time was unacceptably long or when it was considered advantageous to reduce the size of large tumours. Neoadjuvant or adjuvant chemotherapy consisted of cisplatin with 5-fluorouracil, cisplatin with taxoids or cisplatin with both 5-fluorouracil and toxoids, applied every three weeks for two or three cycles. Concurrent chemotherapy consisted of cisplatin given weekly or on weeks 1, 4 and 7 of radiotherapy.

#### Follow-up and statistical analysis

Patient follow-up was measured from the first day of therapy to the day of last examination or death. Patients were examined at least every three months during the first two years, with follow-up examinations every six months thereafter until death. The end points (time to the first defining event) were disease-free survival (DFS), overall survival (OS), loco-regional relapse-free survival (LRRFS) and distant metastasis-free survival (DMFS). DFS was set as the first end point in our current study. Secondary end points were OS, LRRFS and DMFS.

Receiver operating characteristic (ROC) curves was used to calculate the cut-off value of pre-treatment EBV DNA (pre-DNA) based on DFS. Clinical characteristics, including total cholesterol (CHO), triglycerides (TG), low density lipoprotein cholesterol (LDL-C), high density lipoprotein cholesterol (HDL-C), and hypertension, cardiovascular comorbidity were stratified into normal and abnormal groups. Age was classified with the cut-off value of 50 years which was described previously^26^. Pre-DNA was classified according to the cut-off value. The Kruskal-Wallis rank sum test was used to compare differences in pre-treatment plasma EBV DNA levels in different groups. The Chi-square test was used to compare clinical characteristics. Life-table estimation was calculated using the Kaplan-Meier method and differences were compared with the log-rank test. The multivariate Cox proportional hazards model was used to estimate the hazard ratios (HR) and 95% confidence intervals (CI). Variables in the model included age, gender, pathology, T-stage, N-stage, pre-DNA, smoking, drinking, CHO, TG, LDL-C, HDL-C, hypertension, diabetes, cardiovascular comorbidity, and chemotherapy. All statistical tests were two-sided and p < 0.05 was considered statistically significant. The STATA statistical package (STATA 12; StataCorp LP, College Station, Texas, USA) was used for all analyses.

## Results

### Patient baseline characteristics

The clinical characteristics of the 1489 NPC patients are listed in Table 1. In total, 81/1489 (5.4%) were diabetic, 168/1489 (11.3%) were prediabetic, and 1240/1489 (83.3%) were normoglycemic. Of the 81 diabetic patients, 26 (32.1%) received insulin injection alone, 48 (59.3%) received oral antidiabetic drugs alone, and seven (8.6%) received a combination of insulin and oral antidiabetic drugs. There were no significant differences regarding gender, pathology, T-stage, N-stage, smoking, HDL-C, or chemotherapy between diabetic vs. normoglycemic and prediabetic vs. normoglycemic groups. However, diabetic and prediabetic groups contained a higher percentage of patients that were older (*P* < 0.001), hyperlipaemic (*P* < 0.05 for all), suffering from hypertension (*P* < 0.001) and displaying cardiovascular comorbidity (*P* < 0.001) compared with normoglycemic patients. Patients in the diabetic group had lower pre-DNA levels than normoglycemic individuals (*P* = 0.036).

### Failure patterns

The median follow-up time was 49.8 months (range, 1.3–70.7 months), and 227 (15.2%) patients were lost to follow-up. Treatment failure patterns and cause of death are summarized in Table 2. By the last follow-up, 7/81 (8.6%) patients in the diabetic group, 15/168 (8.9%) in the prediabetic group, and 103/1240 (8.3%) in the normoglycemic group experienced locoregional failure, while 11/81 (13.6%) patients in the diabetic group, 16/168 (9.5%) in the prediabetic group, and 133/1240 (10.7%) in the normoglycemic group developed distant metastasis. In addition, 12/81 (14.8%) patients in the diabetic group, 21/168 (12.5%) in the prediabetic group and 117/1240 (9.4%) in the normoglycemic group died, with the majority of deaths attributed to NPC (Table 2).

### Cut-off value of pre-treatment Epstein-Barr virus DNA

Pre-treatment plasma EBV DNA (pre-DNA) was detectable in 54/81 (66.7%) of diabetic patients, 116/168 (69.0%) of prediabetic patients, and 868/1240 (70.0%) of normoglycemic patients. The median pre-DNA was 450 copies/ml (interquartile range, 0–9125) for the diabetic group, 2010 copies/ml (interquartile range, 0–18300) for the prediabetic group, and 1775 copies/ml (interquartile range, 0–14700) for the normoglycemic group. Patients in the diabetic group displayed significantly lower pre-DNA levels than prediabetic or normoglycemic groups (*P* = 0.036). ROC curve analysis of the entire cohort resulted in a pre-DNA cut-off value of 1325 copies/ml based on DFS (AUC, 0.631; sensitivity, 0.648; specificity, 0.591).

### Prognosis of NPC patients with diabetes and prediabetes

**Figure 1:**
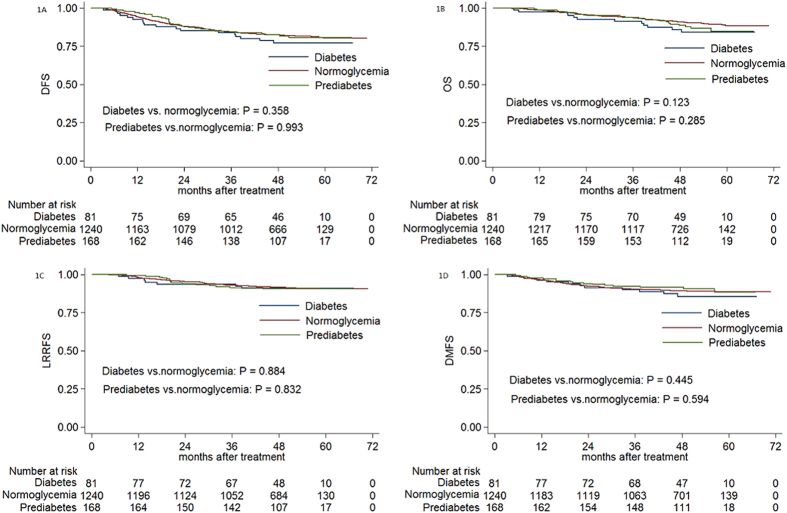
Kaplan-Meier curve analysis of (A) DFS, (B) OS, (C) LRRFS and (D) DMFS in patients with NPC and diabetes, prediabetes or normoglycemia. Abbreviations: DFS = disease-free survival; OS = overall survival; LRRFS = local-regional relapse-free survival; DMFS = distant metastasis-free survival.

**Figure 2:**
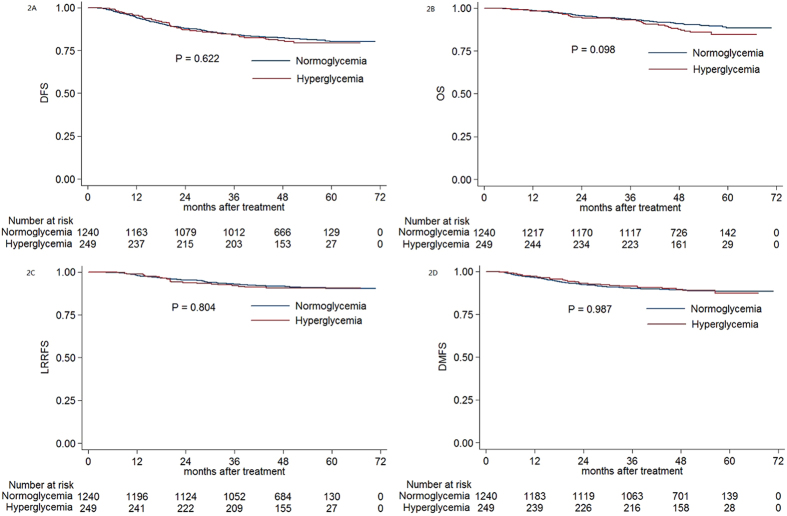
Kaplan-Meier curve analysis of (A) DFS, (B) OS, (C) LRRFS and (D) DMFS in patients with NPC and hyperglycemia, normoglycemia. Abbreviations: DFS = disease-free survival; OS = overall survival; LRRFS = local-regional relapse-free survival; DMFS = distant metastasis-free survival.

The estimated 4-year DFS, OS, LRRFS and DMFS rates for the entire cohort were 82.1%, 90.4%, 91.5% and 89.3%, respectively. The estimated 4-year DFS, OS, LRRFS and DMFS rates were 77.1% vs. 82.4% (*P* = 0.358, Fig. 1A), 85.8% vs. 91.0% (*P* = 0.123, Fig. 1B), 90.9% vs. 91.7% (*P* = 0.884, Fig. 1C), and 85.5% vs. 89.2% (*P* = 0.445, Fig. 1D) for diabetic patients vs. normoglycemic patients, and 82.4% vs. 82.4% (*P* = 0.993, Fig. 1A), 88.7% vs. 91.0% (*P* = 0.285, Fig. 1B), 90.6% vs. 91.7% (*P* = 0.832, Fig. 1C) and 91.5% vs. 89.2% (*P* = 0.594, Fig. 1D) for preidabetic patients vs. normoglycemic patients. When diabetic and prediabetic groups were combined, no significant difference was found compared with the normoglycemic group (*P* = 0.622 for DFS, *P* = 0.098 for OS, *P* = 0.804 for LRRFS, *P* = 0.987 for DMFS; Fig. 2).

Univariate analysis found no significant difference in 4-year DFS, OS, LRRFS and DMFS in diabetic patients vs. normoglycemic patients or prediabetic patients vs. normoglycemic patients. Multivariate analysis (Table 3) was performed to adjust for various prognostic factors. After adjustment for age, gender, pathology, T-stage, N-stage, pre-DNA, smoking, drinking, CHO, TG, LDL-C, HDL-C, hypertension, cardiovascular complications and chemotherapy, no significant differences in 4-year DFS, OS, LRRFS and DMFS were found between diabetic vs. normoglycemic and prediabetic vs. normoglycemia groups.

### Subgroup analysis based on clinical stage and pre-DNA levels

We next assessed the prognostic value of diabetes in patients with early-stage and locoregional advanced NPC (Table 4). In patients with stage III/IV NPC, no significant difference was found between diabetic and normoglycemic and prediabetic and normoglycemic groups (*P* > 0.05 for all rates). Among patients with stage I/II disease, a significant difference was only observed for OS between prediabetes and normoglycemia groups (P < 0.001). However, multivariate analysis did not identify diabetes as an independent prognostic factor for DFS (*P* = 1.00; HR, 1.00; 95% CI, 0.697–1.434), OS (*P* = 0.087; HR, 1.495; 95% CI, 0.943–2.369), LRRFS (*P* = 0.521; HR, 1.169; 95% CI, 0.726–1.882) and DMFS (*P* = 0.408; HR, 1.195; 95% CI, 0.784–1.823).

Subgroup analysis was also conducted in patients with pre-DNA levels >1325 copies/ml (Pre-H) and pre-DNA levels <1325 copies/ml (Pre-L). In pre-H patients, no statistical significance was observed for 4-year DFS, OS, LRRFS and DMFS rates (*P* > 0.05 for all rates; Table 4). Among pre-L patients, a significant difference was only observed in DFS (81.1% vs. 91.0%, *P* = 0.036), OS (85.1% vs. 96.4%, *P* < 0.001) and DMFS (89.2% vs. 95.7%, *P* = 0.044) between diabetes and normoglycemia groups. However, multivariate analysis did not identify diabetes as an independent prognostic factor for DFS (*P* = 0.338; HR, 1.167; 95% CI, 0.851–1.602), OS (*P* = 0.17; HR, 1.330; 95% CI, 0.885–1.998), LRRFS (*P* = 0.218; HR, 1.289; 95% CI, 0.861–1.930) and DMFS (*P* = 0.861; HR, 1.045; 95% CI, 0.637–1.714).

## Discussion

To the best of our knowledge, this study is the largest to investigate the impact of diabetes on the prognosis of non-metastatic NPC patients treated using uniform IMRT. This work is particularly relevant to populations such as south China that have a high prevalence of NPC. However, our results indicate that diabetes is not an independent prognostic factor for non-metastatic NPC patients receiving IMRT. The incidence of diabetes in our cohort was 5.4%, which was similar to that of a similar previous study (5.9%)^21^. In contrast, the incidence of prediabetes in our cohort (11.3%) was far lower than this previous study (26.7%)^21^. This apparent discrepancy could be explained by the more detailed data analysis and stricter patient selection criteria employed in the present work.

Various potential mechanisms linking diabetes to carcinogenic processes have been reported, including untreated hyperglycemia providing more energy for tumour growth and neoplastic proliferation^11^. In diabetic patients, hyperglycemia results in the mitochondrial electron transport chain overproducing superoxide and reactive oxygen species^9^,^27^. Most cancer cells express insulin and insulin-like growth factor (IGF), which could stimulate insulin-mediated mitogenesis and cancer cell proliferation and metastasis^11^. In addition to the direct effects of insulin and IGF, the inflammatory cytokines produced by adipose tissue may stimulate malignant progression^11^. Numerous studies have reported a significant association between hyperglycemia and the survival of patients with lung, pancreatic and breast cancers^15^,^16^,^17^,^18^. However, our current study did find a correlation between negative outcomes of patients with NPC and hyperglycemia. This may be due to differences in the biology and pathology of NPC vs. lung and breast cancers. In addition, the study by Liu et alAlternatively, concurrent chemoradiotherapy and better management of diabetes patients may have improved their survival outcomes, thereby masking diabetes as a prognostic factor.

Pre-DNA levels are a reliable molecular marker for guiding treatment and predicting prognosis^4^,^5^,^22^. Patients with newly diagnosed diabetes are reported to have an increased risk of NPC^19^, but the relationship between plasma EBV DNA and diabetes was not considered in this earlier study. However, in the present study, patients with diabetes were found to have significantly lower pre-DNA levels compared with the normoglycemia group. Even after adjustment for these factors, diabetes and prediabetes did not have prognostic value for 4-year DFS, OS, LRRFS and DMFS, and multivariate analysis of subgroups based on Pre-H and Pre-L did not change this. These results suggest there is no correlation between EBV and diabetes in patients with NPC.

Diabetes can cause glucose-mediated vascular damage that may result in comorbidities such as blindness, renal failure, nerve damage, limb amputation, myocardial infarction and stroke^27^. In our present study, patients with diabetes or prediabetes were associated with a higher prevalence of hyperglycemia-related comorbidities such as hypertension, cardiovascular complications and hyperlipaemia. We previously showed that comorbidity significantly affect the prognosis of NPC patients, especially those in stages II and III^28^. Additionally, Kiderlen *et al.* (2013) found that patients with diabetes but no other comorbidity had a similar overall survival outcome to patients without any comorbidities^18^. This is a reminder that diabetes-associated comorbidities play an important role in the prognosis of NPC patients in addition to diabetes itself. Consistent with this, multivariate analysis in the present study identified hyperlipaemia as an independent prognostic factor for DFS, OS, LRRFS and DMFS. Patients with diabetes-related hyperlipaemia exhibited poor physical condition, resulting in poor prognosis, suggesting more attention should be paid to NPC patients with diabetes and diabetes-related hyperlipaemia.

Of note, diabetes is a kind of chronic disease, and it takes many years to develop these comorbidities which influence the prognosis. Therefore, for newly diagnosed diabetic patients, the median follow-up of 4 years may be not enough to show poor influence of diabetes. Thus, it may be reasonable to exclude these newly diagnosed diabetic patients when evaluating the prognostic value within such relatively insufficient follow-up time, and future studies should take good care of this.

To the best of our knowledge, this is the first study to assess the relationship between diabetes and prognosis of NPC patients treated using IMRT. Plasma EBV DNA levels of NPC patients with diabetes and prediabetes were investigated, but no definitive link to NPC was made. The present study is limited by its retrospective nature and potentially insufficient follow-up time, although we did select DFS as the major endpoint to address this shortcoming. A longer follow-up time may be needed in future studies addressing the prognostic value of diabetes. Glycated haemoglobin (HbA1c) was recently reported to be a more reliable marker diabetes management^29^, and this could be incorporated into any future research.

## Conclusion

In our current study, diabetes was found to have no prognostic impact on NPC patients treated using IMRT, even after adjustment for plasma EBV DNA and other prognostic factors. However, TG and LDL-C did have a poor impact on 4-year DFS, OS, LRFFS and DMFS. In all, our results suggest more attention should be paid to hyperglycemia-related hyperlipaemia in NPC patients with diabetes.

## Additional Information

**How to cite this article**: Peng, H. *et al.* Prognostic value of Diabetes in Patients with Nasopharyngeal Carcinoma Treated with Intensity-Modulated Radiation Therapy. *Sci. Rep.* **6**, 22200; doi: 10.1038/srep22200 (2016).

## Figures and Tables

**Table 1:** Baseline characteristics of NPC patients with diabetes and prediabetes.

Characteristics	Normoglycemia	Diabetes	P_1_	Prediabetes	P_2_
	No. (%)	No. (%)		No. (%)	
Total	1240	81		168	
Age (years)			<0.001		<0.001
≥50	354 (28.5)	53 (65.4)		94 (56.0)	
<50	886 (71.5)	28 (34.6)		74 (44.0)	
Gender			0.561		0.176
Male	914 (73.7)	64 (79.0)		132 (78.6)	
Female	326 (26.3)	17 (21.0)		36 (21.4)	
WHO pathology			0.451		0.329
Type I	7 (0.6)	1 (1.2)		0 (0)	
Type II/III	1233 (99.4)	80 (98.8)		168 (100)	
T classification^a^			0.2		0.724
T1 + T2	423 (34.1)	22 (27.2)		55 (32.7)	
T3 + T4	817 (65.9)	59 (72.8)		113 (67.3)	
N classification^a^			0.423		0.319
N0 + N1	937 (75.6)	58 (71.6)		121 (72.0)	
N2 + N3	303 (24.4)	23 (28.4)		47 (28.0)	
Overall stage^a^			0.501		0.443
I	70 (5.6)	2 (2.4)		5 (3.0)	
II	259 (20.9)	14 (17.3)		40 (23.8)	
III	586 (47.3)	42 (51.9)		81 (48.2)	
IVA-IVB	325 (26.2)	23 (28.4)		42 (25.0)	
Pre-DNA (copy/ml)			0.036		0.434
≥1325	654 (52.7)	33 (40.7)		94 (56.0)	
<1325	586 (47.3)	48 (59.3)		74 (44.0)	
Smoking			0.344		0.734
Yes	463 (37.3)	26 (32.1)		65 (38.7)	
No	777 (62.7)	55 (67.9)		103 (61.3)	
Drinking			0.195		0.009
Yes	170 (13.7)	7 (8.6)		11 (6.5)	
No	1070 (86.3)	74 (91.4)		157 (93.5)	
CHO			0.006		<0.001
≥6.47	113 (9.1)	15 (18.5)		30 (17.9)	
<6.47	1127 (90.9)	66 (81.5)		138 (82.1)	
TG			0.001		<0.001
≥1.70	368 (29.7)	41 (50.6)		75 (44.6)	
<1.70	872 (70.3)	40 (49.4)		93 (55.4)	
LDL-C			0.03		0.007
≥3.40	521 (42.0)	44 (54.3)		89 (53.0)	
<3.40	719 (58.0)	37 (45.7)		79 (47.0)	
HDL-C			0.518		0.336
≥0.78	1211 (97.7)	80 (98.8)		162 (96.4)	
<0.78	29 (2.3)	1 (1.2)		6 (3.6)	
Hypertension			<0.001		<0.001
Yes	86 (6.9)	24 (29.6)		28 (16.7)	
No	1154 (93.1)	57 (70.4)		140 (83.3)	
Cardiovascular complications			<0.001		<0.001
Yes	101 (8.1)	25 (30.9)		30 (17.9)	
No	1139 (91.9)	56 (69.1)		138 (82.1)	
Chemotherapy			0.714		0.647
Yes	1069 (86.2)	71 (87.7)		147 (87.5)	
No	171 (13.8)	10 (12.3)		21 (12.5)	

Abbreviations: WHO = World Health Organization; Pre-DNA = pre-treatment Epstein-Barr virus DNA; CHO = total cholesterol; TG = triglycerides; LDL-C = low density lipoprotein cholesterol; HDL-C = high density lipoprotein cholesterol.

^a^According to the 7^th^ edition of the AJCC/UICC staging system.

P_1_: diabetes vs. normoglycemia; P_2_: prediabetes vs. normoglycemia.

**Table 2:** Treatment failure patterns and causes of death.

Failure pattern	Normoglycemia	Diabetes	P_1_	Prediabetes	P_2_
	No. (%)	No. (%)		No. (%)	
Local only	43 (20.7)	3 (17.6)	0.847	4 (14.8)	0.466
Local + regional	8 (3.8)	0 (0)	1.000	3 (11.1)	0.276
Local + distant	10 (4.8)	1 (5.9)	0.504	2 (7.4)	0.939
Local + regional + distant	6 (2.9)	0 (0)	1.000	1 (3.7)	0.590
Regional only	24 (11.5)	3 (17.6)	0.465	4 (14.8)	0.944
Regional + distant	12 (5.8)	0 (0)	1.000	1 (3.7)	0.970
Distant only	105 (50.5)	10 (58.8)	0.224	12 (44.4)	0.574
Total locoregional	103 (49.5)	7 (41.2)	0.916	15 (55.6)	0.785
Total distant	133 (63.9)	11 (64.7)	0.424	16 (59.3)	0.635
Total failure	208	17		27	
Causes of Death			1.000		0.675
Cancer	102 (87.2)	11 (91.7)		17 (81.0)	
Non-cancer	15 (12.8)	1 (8.3)		4 (19.0)	
Total	117	12		21	

Abbreviations: P_1_: diabetes vs. normoglycemia; P_2_: prediabetes vs. normoglycemia.

**Table 3:** Multivariate analysis of prognostic factors correlated with clinical outcomes.

Endpoint	Variable	P^a^	HR	95% CI for HR
DFS	Age	0.021	1.334	1.044–1.704
	Pathology	0.042	0.355	0.131–0.961
	N-classification	<0.001	1.712	1.329–2.206
	LDL-C	0.045	1.279	1.005–1.626
	Pre-DNA	<0.001	2.202	1.674–2.897
OS	Age	<0.001	1.951	1.414–2.693
	T-classification	0.001	2.043	1.337–3.122
	N-classification	<0.001	2.177	1.558–3.043
	LDL-C	0.026	1.445	1.045–1.998
	Pre-DNA	0.002	1.836	1.252–2.693
LRRFS	Gender	0.042	1.493	1.015–2.195
	Pathology	<0.001	0.102	0.037–0.278
	TG	0.043	1.457	1.012–2.096
	Pre-DNA	<0.001	1.958	1.351–2.839
DMFS	N-classification	<0.001	2.295	1.665–3.163
	LDL-C	0.034	1.400	1.025–1.910
	Pre-DNA	<0.001	2.897	1.959–4.284

Abbreviations: DFS = disease-free survival; OS = overall survival; LRRFS = loco-regional relapse-free survival; DMFS = distant metastases-free survival; HR = hazard ratio; CI = confidence interval; Pre-DNA = pre-treatment Epstein-Barr virus DNA; TG = triglycerides; LDL-C = low density lipoprotein cholesterol; a: Multivariate *P*-values were calculated using an adjusted Cox proportional-hazards model with backward elimination and the following parameters: age (≥50 vs. <50), gender (male or. female), pathology (Type I or Type II/III), T-classification (T_3–4_ or T_1–2_), N-classification (N_2–3_ or N_0–1_), pre-DNA (≥1325 copies/ml vs. <1325 copies/ml), smoking (yes or no), drinking (yes or no), CHO (≥6.47 vs. <6.47), TG (≥1.70 vs. <1.70), LDL-C (≥3.40 vs. <3.40), HDL-C (≥0.78 vs. <0.78), hypertension (yes or no), cardiovascular complications (yes or no), use of chemotherapy (yes or no).

**Table 4:** Subgroup analysis based on clinical stage and pre-DNA levels.

	Stage I–II				Stage III–IV				Pre–L				Pre–H			
	DFS	OS	LRRFS	DMFS	DFS	OS	LRRFS	DMFS	DFS	OS	LRRFS	DMFS	DFS	OS	LRRFS	DMFS
Diabetes	93.8	93.8	100	93.8	72.9	83.7	88.5	83.3	81.1	85.1	91.5	89.2	71.1	86.6	90.0	80.2
Prediabetes	82.0	93.1	86.4	91.1	82.6	87.0	92.3	91.7	85.5	91.9	90.2	95.9	80.5	85.7	91.1	88.0
Normoglycemia	90.1	99.1	93.7	95.1	79.6	88.0	90.9	87.0	91.0	96.4	95.1	95.7	74.8	86.1	88.4	83.4
P_1_	0.601	0.089	0.295	0.068	0.325	0.309	0.584	0.289	0.036	<0.001	0.345	0.044	0.926	0.776	0.708	0.872
P_2_	0.162	<0.001	0.13	0.097	0.529	0.944	0.534	0.213	0.207	0.094	0.177	0.724	0.377	0.862	0.464	0.347

Abbreviations: DFS = disease-free survival; OS = overall survival; LRRFS = loco-regional relapse-free survival; DMFS = distant metastases-free survival.

Pre-L = pre-treatment Espstein-Barr virus DNA <1325 copies/ml; Pre-H = pre-treatment Epstein-Barr virus DNA ≥1325 copies/ml.

P_1_: diabetes vs. normoglycemia; P_2_: prediabetes vs. normoglycemia.

Unless otherwise indicated, numbers are percentages.
